# Circulating Endometrial Cells: A New Source of Information on Endometriosis Dynamics

**DOI:** 10.3390/jcm8111938

**Published:** 2019-11-11

**Authors:** Eliska Pospisilova, Imrich Kiss, Helena Souckova, Pavel Tomes, Jan Spicka, Rafal Matkowski, Marcin Jedryka, Simone Ferrero, Vladimir Bobek, Katarina Kolostova

**Affiliations:** 1 Department of Laboratory Genetics, Laboratory Diagnostics, Faculty Hospital Královské Vinohrady, Srobarova 50, 100 34 Prague 10, Czech Republicvbobek@centrum.cz (V.B.); 2 Department of Gynecology, Military University Hospital and 3rd Faculty of Medicine, U Vojenske nemocnice 1200, 169 02 Prague 6, Czech Republic; 3 Department of Obstetrics and Gynecology, University Hospital, Faculty of Medicine Charles University, Alej Svobody 80, 301 66 Pilsen, Czech Republic; 4 Cellpeutics Sp. z o.o., Duńska 9,54-424 Wrocław, Poland; 5 Department of Oncology, Wroclaw Medical University, Wybrzeże Ludwika Pasteura 1, 50-367 Wrocław, Poland and Wroclaw Comprehensive Cancer Center, Plac Ludwika Hirszfelda 12, 53-413 Wrocław, Poland; 6 Academic Unit of Obstetrics and Gynecology Ospedale Policlinico San Martino Genoa, Italy Department of Neurosciences, Rehabilitation, Ophthalmology, Genetics, Maternal and Child Health (DiNOGMI) University of Genoa Genoa, Italy Academic Unit of Gynecology and Obstetrics University of Genoa, 16 132 Genoa, Italy; 7 Department of Histology and Embryology, Wroclaw Medical University, L. Pasteur 1, 503 67 Wroclaw, Poland; 8 Department of Thoracic Surgery, Krajská zdravotní a.s. Hospital, 41100 Ústí nad Labem, Czech Republic; 9 3rd Department of Surgery University Hospital FN Motol and 1st Faculty of Medicine Charles University, V Uvalu 84, 150 06 Prague 5, Czech Republic

**Keywords:** circulating endometrial cells, endometriosis, rare cells, menstrual cycle, liquid biopsy

## Abstract

The focus of the presented work was to isolate and characterize circulating endometrial cells (CECs) enriched from peripheral blood (PB) of patients with diagnosed endometriosis. The molecular characteristics of CECs could be supportive for an understanding of endometriosis pathogenesis and treatment decisions in the future. Material and Methods: Blood samples (*n* = 423) were tested for CECs presence. Subsequently, gene expression analysis (GEA) was carried out for CECs. In parallel, CECs presence and characteristics were tested during menstrual cycle (MC) phases in 11 patients. CECs were enriched by size-based separation. Results: CECs were present in 78.4% of the tested blood samples. In line with the revised American Fertility Society (rAFS) classification, CECs presence was confirmed in all the acknowledged endometriosis stages: minimal, mild, moderate, and severe. Surprisingly, CECs negativity rate was also reported for severe disease in 21.1% of cases. The CECs captured during MC phases displayed different cytomorphology, including epithelial, stromal, and stem cell-like characteristics. The highest CECs numbers were detected in the mid-secretory phase of MC, which corresponds to uterine lining decidualization. CECs captured during mid-secretory periods expressed genes *KRT18*, *NANOG*, and *VIM* in higher amounts when compared to the proliferative phase of MC, where genes *KRT19* and *ESR1* were mostly elevated. GEA of the super-positive CECs samples (1000 CECs/8 mL PB) revealed high expression of genes *KRT18*, *VIM*, *NANOG*, and *FLT1*. The expression of these genes was also elevated in the endometriosis tissue samples and endometrioma. Conclusion: The panel of the identified CEC genes could be tested in a prospective manner to confirm the role of CECs in endometriosis pathogenesis and diagnostics.

## 1. Introduction

Endometriosis is a common disease among women of reproductive age and a major contributor to pelvic pain and subfertility causing disability and significantly compromised quality of life [1]. It affects up to 10% of women of reproductive age, 50–60% of women and two-thirds of teenage girls with pelvic pain and dysmenorrhea, and up to 50% of women with infertility [2,3]. Because of its wide and non-specific clinical symptoms and difficult diagnosis, endometriosis is frequently underdiagnosed or diagnosed in later, more severe stages [4]. Diagnostic lead marks could be put together from the patient’s history, gynecological examination containing ultrasound, and a few specific laboratory markers such as CA125 (cancer antigen 125, known as MUC16). Until now, there is no biomarker from the endometrium, blood, or urine or combined non-invasive tests specific enough to be used in clinical practice. Therefore, laparoscopy remains the gold standard for the diagnosis of endometriosis, and using non-invasive tests should only be undertaken in a research setting [1,5,6,7,8].

As the etiology and pathophysiology of endometriosis is still not fully understood, and more theories are being studied, it is challenging to discover a highly specific and sensitive preoperative diagnostic tool. Furthermore, it is necessary to validate the diagnostic accuracy of every promising test prospectively in an independent symptomatic patient population with subfertility and/or pain without clear ultrasound evidence of endometriosis and with a clinical indication for surgery, divided into cases with laparoscopically and histologically confirmed endometriosis and controls with laparoscopically confirmed absence of endometriosis [9].

To ensure a full understanding of the hypothesis of circulating endometrial cells (CECs), the lymphovascular spread (also called embolization, metastasis, transplantation) theory must be introduced first. It was first published by Halban in 1925 [10], who detected endometrial cells in the lymphatic system of the uterus in patients with endometriosis. Meanwhile, Sampson studied the volume and shape of the uterine cavity in normal and pathologic conditions. When injecting the uterus with a suspension, he found the injected mass to escape from uterine veins, which led him to believe that the endometrial cells would enter circulation in the same way [11]. This theory was further studied in an experiment in 1940, when Hobbs and Bortnick injected endometrial cells into the circulation of rabbits. He found endometrial lesions in the lungs and pleura of these animals later during dissection [12]. In 1952, Javert followed up on Sampson’s work and detected endometrial cells in the pelvic veins of patients with endometriosis [13].

More than half a century later, CECs were described by Bobek et al. [14] in accordance with the same vascular spread theory. Endometrial cells from peripheral blood (PB) and peritoneal washings (PW) in patients with endometriosis were successfully isolated by a size-based separation method (Metacell^®^). The endometrial origin of the captured cells was proven by immunohistochemistry [14]. Later, Chan et al. used immunofluorescence staining and separation via microfluidic chips for CEC detection. The results indicated that CECs could be a promising biomarker with great potential in the diagnosis of endometriosis [15].

The aim of this study was to isolate CECs in patients with different types of endometriosis and clinical symptoms and to characterize these cells by molecular analysis. In agreement with the innovative stem cell-based concept of endometriosis origin [16], CECs molecular analysis might fulfil the puzzle of endometriosis pathogenesis.

The results of CECs cytomorphological analysis and gene expression profiling were correlated with patients’ clinical data. Further analysis was conducted in patients with multiple sampling throughout the menstrual cycle (MC) to understand the characteristics of CECs in each phase of MC. New information on the possible pathophysiology and development of endometriosis was brought through comparisons of molecular profiles of endometrial lesions obtained during gynecological surgeries and CECs.

## 2. Material and Methods

A multi-center prospective study was initiated to collect blood samples from women with endometriosis. The inclusion criterion was that all the patients had a histologically proven history of endometriosis. A form was filled for each patient containing her detailed data, i.e., information about the menstruation cycle, hormonal therapy if any, type of endometriosis (ovarian, peritoneal, recto-vaginal septum, adenomyosis, extragenital), classification by revised American Fertility Society (rAFS), symptoms, and signs (pelvic pain, dysmenorrhea, dyspareunia, metrorrhagia, hypermenorrhea, sterility, infertility, gastrointestinal problems). A supplementary table reporting the clinical data is available. Samples were obtained from 423 patients. The protocol for this study was approved by the Ethical Committee of University hospital Kralovske Vinohrady in Prague, Czech Republic (EK-VP/20/0/2015) and the Ethical Committee of Medical University Wroclaw, Poland (Nr.KB-242/2015 part of study and grant SUB.C280.19.050).

As the samples were collected from various centers nationally and internationally, a possible bias in results due to different transport conditions was considered. The sample was marked CEC-positive if endometrial-like cells were detected. Subsequently, we divided the positive samples into categories based on CEC quantity (low positivity—up to 10 cells, medium positivity—up to 100 cells, high positivity—more than 100 cells). Samples with high positivity (*n* = 13) were subjected to molecular analysis. To be able to analyze the molecular character of CECs during the MC, we obtained multiple samples from 11 patients during their menstrual cycle (2 × 48 samples in total). A minimum of four samples were taken for every patient to correspond to different phases of the cycle (menstruation, proliferative phase, ovulation, secretory phase). The phase was calculated from the last menstrual bleeding and verified by ultrasound examination of the endometrium.

To enrich CECs, approximately 2 × 8 mL of PB was drawn from the antecubital veins and placed into S-Monovette tubes (Sarstedt AG & Co., Numbrecht, Germany) containing 1.6 mg EDTA/mL blood as an anticoagulant. The samples were processed at room temperature using an isolation procedure completed within 36 h of the blood draw. The ethics committees of the participating universities and hospitals approved the study protocol according to the Declaration of Helsinki. Size- based filtration and an in vitro culture method (MetaCell^®^, Ostrava, Czech Republic) were used to enrich CECs. [14]. The captured cells grew in the fetal bovine serum -enriched RPMI medium (10%) (Merck KGaD, Darmstadt, Germany) for the period of a minimum of 7–14 days on the separation membrane. The cultured cells were analyzed by vital fluorescent microscopy using unspecific nuclear (NucBlue^TM^_,_ ThermoFisherScientific, Waltham, U.S.) and cytoplasmatic (Celltracker^TM^_,_ ThermoFisherScientific, Waltham, U.S.) staining. Cells on the membrane were later put into RLT buffer lysis (Qiagen, Hilden, Germany) and kept in the freezer for further analysis.

As mentioned earlier, further molecular analysis was initiated in the single site sample group with the highest number of CECs (2 × 13 samples in total). To confirm the origin of the cells on the separation membrane, CECs gene expression analysis was performed. Gene expression analysis (GEA) allowed up to 20 endometriosis-associated markers in RNA from different cell fractions to be tested within a single quantitative polymerase chain reaction (qPCR) run. Differential diagnostic markers for the qPCR test were chosen in concordance with the expected diagnosis. The key purpose of GEA was to compare gene expression of endometriosis-associated markers in the CECs enriched fractions to that in the whole blood.

Soon after, RNA was isolated from the whole blood’s white blood cell fraction (WBC) and CEC-enriched fraction on the membrane. Finally, the CECs gene expression analysis allowed identification of the relative amount of endometriosis-associated markers in the whole blood and in CEC-enriched fractions. The RNA from the whole blood was isolated with a modified procedure, and the quality/concentration of RNA was measured by NanoDrop (ThermoFisherScientific, Waltham, U.S.). As there were only a few hundred cells on the membrane, the median concentration of RNA was quite low (5–10 ng/μL). A High-Capacity complementary DNA (cDNA) Reverse Transcription Kit (ThermoFisherScientific, Waltham, U.S.) was used for cDNA production. qPCR analysis was performed using Taqman chemistry with hydrolysis probes for all the tested genes (ThermoFisherScientific, Waltham, U.S.). The tested genes which were thought to be endometrial-associated were *CD68*, *EpCAM*, *KRT7*, *KRT18*, *KRT19*, *MUC1*, *MUC16*, *VIM*, *VEGFA*, *WT1*, *ESR1*, *PGR*, *HER2*, *CD10*, *FLT1*, *MMP1*, *MMP9*, *TP63*, *ESSRA*, *ESSRB*, *HIF1A*, and *NANOG*.

To ectopically analyze growing endometrial cells in tissues, we obtained several layers of histologically proven endometrioma (*n* = 11) from two patients during gynecological surgeries. Both patients had procedures planned because of pelvic pain and had a cystic adnexal tumor diagnosed during ultrasound examination. The perioperative findings in the first patient were bilateral massive endometriomas of the ovaries with no peritoneal or other lesions. The second patient had one-sided endometrioma forming a convolute consisting of the ovary and fallopian tube, severe peritoneal lesions of the urinary bladder, sacrouterine ligaments, and Douglas pouch. Eutopic endometrial tissue during menstruation bleeding was acquired from a healthy control. All tissues were further analyzed using the same qPCR protocol as for the CECs samples.

Gene expression analysis was conducted using Genex v. 6 (MultiD, Sweden) software to enable normalization and statistical analysis (cluster analysis, Mann–Whitney tests) for qPCR-generated data. The relative RNA amounts are reported for tested groups in comparisons to white blood cell fractions (WBC) or endometriosis tissue.

## 3. Results

### 3.1. CECs Presence in Endometriosis

**Figure 1:**
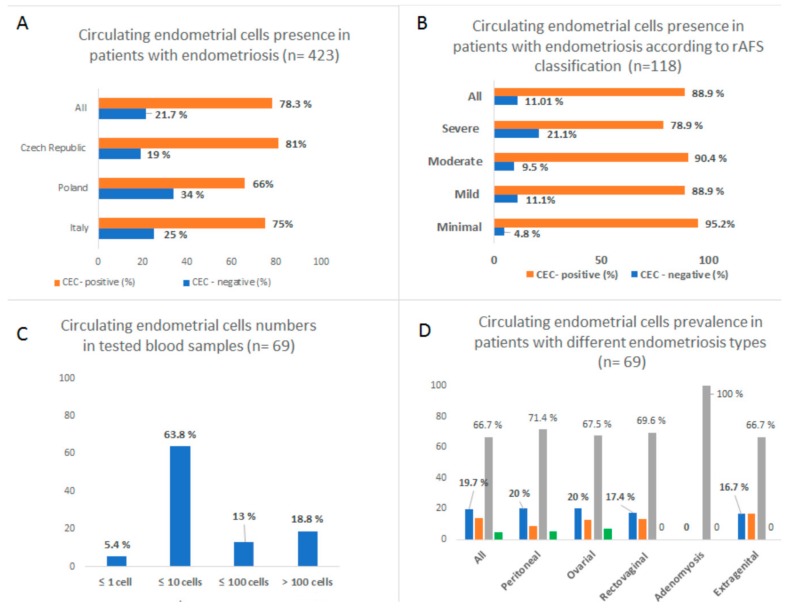
Circulating endometrial cells (CECs) presence in patients with endometriosis. (**A**) CEC-positive/negative sample frequency is shown for all of the tested patients. (**B**) CEC-positive/negative sample frequency is shown in connection to the revised American Fertility Society (rAFS) classification. (**C**) CEC prevalence/load in tested samples is shown as numbers counted. The numbers of CECs were then ascribed to the following categories: CEC-negative (≤1 cell detected) and CEC-positive: 1–10 cells (low positivity), 10–100 cells (medium positivity), and >100 cells (high positivity). The distribution of CECs load in the tested samples reflected normal distribution in the tested cohorts. (**D**) CECs load is shown for different endometriosis types.

CECs were detected by cytomorphological evaluation in 78.3% (331/423) of the tested samples. Four main CEC subtypes can be found in blood sample of patients with endometriosis: epithelial, stem cell-like, stromal, and glandular. CEC positivity did not vary significantly in different patient cohorts from Italy (*n* = 20), Poland (*n* = 82), or Czech Republic (*n* = 321) (75% vs. 66% vs. 81%) (see Figure 1A).

In line with the rAFS classification, CECs presence was confirmed for all of the acknowledged endometriosis stages: minimal, mild, moderate, and severe. Surprisingly, there was a significant portion of CEC-negative samples reported for severe disease (21.1%) (see Figure 1B).

### 3.2. CECs Load in Patients with Different Endometriosis Types

If accessible, CECs numbers were counted and then ascribed to the following categories: CEC-negative (≤1 cell detected) and CEC-positive: 1–10 cells (low positivity), 10–100 cells (medium positivity), and >100 cells (high positivity) (see Figure 1C–D). The distribution of CEC load in the tested samples reflected normal distribution in the tested cohorts. The conclusion was that, in 20% of cases, there were patients with very high CEC numbers, and, in 10–20% of patients with endometriosis, there were no CECs present in PB.

The highest CEC numbers were detected in the after-ovulation periods (day 14–17, i.e., secretory phase), which corresponds to estrogen decrease and slow subsequent progesterone increase associated with uterine lining decidualization. We succeeded in setting up in vitro cultures of isolated cells.

For ovarian, peritoneal, rectovaginal, and extragenital endometriosis, CECs were found in 90–95% of samples and mostly in numbers of 1–10 cells (low positivity) for 8 mL of PB (see Figure 1D). It was confirmed that CEC presence is most probably independent of the different extrauterine (ectopic) locations of endometriosis tissue. Finally, it was shown that there is a subgroup of patients in all of the mentioned endometriosis subgroups with very high numbers of CECs (up to 20% of patients) in the blood.

### 3.3. Gene Expression Profiling of Endometriosis Tissue and Related CECs Samples

To confirm the origin of CECs in PB evaluated by cytomorphology, additional molecular testing was provided, analyzing gene expression of endometriosis tissue samples (TS) and blood samples from the same patient (WBC, CECs). Up to 20 markers were analyzed in total. The cluster analysis of normalized qPCR results enabled identifying a group of “endometriosis” genes that could be used as confirmatory for CECs.

**Figure 2:**
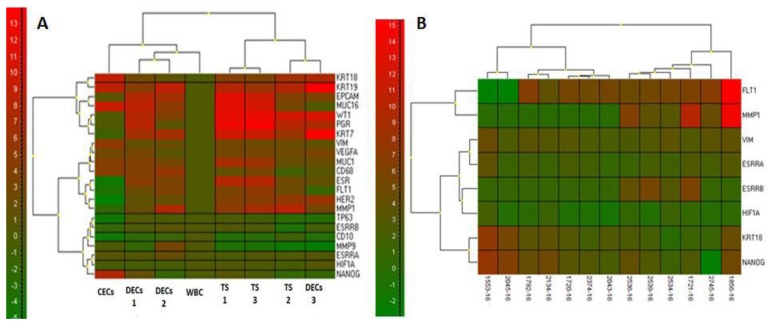
Relative RNA quantity for CECs and endometriosis tissue samples shown after cluster analysis. (**A**) Samples for one patient’s CEC tissue sample (TS) collection are presented with eight samples in total. Tissue samples TS 1–3 were obtained during surgery, as well as peritoneal washing (PW; a source for disseminated endometrial cells (DEC)). DEC 1 and DEC 2 were cultured after size-based filtration. DEC 3 was cultured without preliminary enrichment of PW. In the left subcluster, there are gene expression profiles of loose endometrial cells from the blood and DECs isolated by size-based separation. The second cluster is represented by endometriosis tissue biopsy samples TS 1, TS 2, and TS 3 and in vitro cultured PW. Two tissue samples (TS 1 and TS 3) are clustered together and show a very high level of similarity. For this patient with an endometriotic cyst, *MUC16* was also detected on CECs. (**B**) Cluster analysis for high-positive CECs samples is shown. The analysis revealed that there were at least two different sample types of high-positive CECs (two clusters). The first one is represented by the cluster showing a high expression of *FLT1*, *MMP1*, and *ESRRB* (cluster on the right). The second group of samples shows elevation of *NANOG*, *KRT18*, and *VIM* expression. *KRT19* was relatively highly expressed in both clusters (not shown).

There were several genes strongly expressed in endometriosis tissue: *EPCAM*, *KRT18*, *WT1*, *MUC16*, *MUC1*, and *ESR1*, if compared to the endometrial cells from healthy controls. In some tissue samples, *MMP1* was also present. In the CEC fraction of these patients, only *KRT18*, *KRT19*, *VIM*, and *NANOG* were detected in a relatively high amount compared to the WBC profile. Interestingly, in CEC samples, *ESR1* was expressed very rarely. The genes with increased expression (*KRT18*, *KRT19*, *VIM*, and *NANOG*) were then used in subsequent analyses as “endometriosis confirmatory genes”. The cluster analysis of qPCR results for a sample collection of one patient is shown in Figure 2A.

### 3.4. Gene Expression Analysis of Positive CECs Samples with Significant Cellularity

The genes confirmed to be expressed in the endometriosis tissue and CECs, as presented in the first part of the results (Figure 2A), were then analyzed in the group of patients (*n* = 13) in whom CECs were detected in relatively high numbers (up to 1000 CECs/8 mL PB). In this CEC cohort, the following genes were confirmed to be elevated: *VIM* (elevated in 13 out of 13 CECs samples—13/13), *FLT1* (12/13), *KRT18* (8/13), *KRT19* (8/13), *MMP9* (12/13), *NANOG* (8/13), and *ESR1* (7/13). The statistical significance of differential expression values was confirmed for *VIM*, *MMP9*, *FLT1*, and *KRT19*. As expected, CD68 was elevated in all of the tested samples (13/13), which suggests that some of the frequently observed genes could be found because of the presence of the captured and in vitro cultured macrophages, which have a very similar cytomorphology to the endometrial cells. However, correlation analysis revealed that *KRT18*, *FLT1*, and *NANOG* expression was *CD68*-independent. On the other hand, in this specific patient’s cohort, there was a correlation between *CD68* and *VIM*.

The cluster analysis of these positive CEC samples revealed that there were at least two different cell types of CECs in the analyzed samples. The first one was represented by the cluster showing a high expression of *FLT1*, *MMP1*, and *ESRRB* (see Figure 2B—cluster on the right). The second group of samples showed an elevation of *NANOG*, *KRT18*, and *VIM* expression. *KRT19* was relatively highly expressed in both clusters (not shown). Significantly elevated *FLT1* did not correlate with any other of the tested genes. Interestingly, *ESR1* expression was present in parallel with *ESSRA* and *ESSRB* in two super-positive samples only. Very high expression of *ESSRB* was observed in samples with elevated *VIM* expression.

### 3.5. Gene Expression Profiling Data of CEC—Samples with Average Cellularity

**Figure 3:**
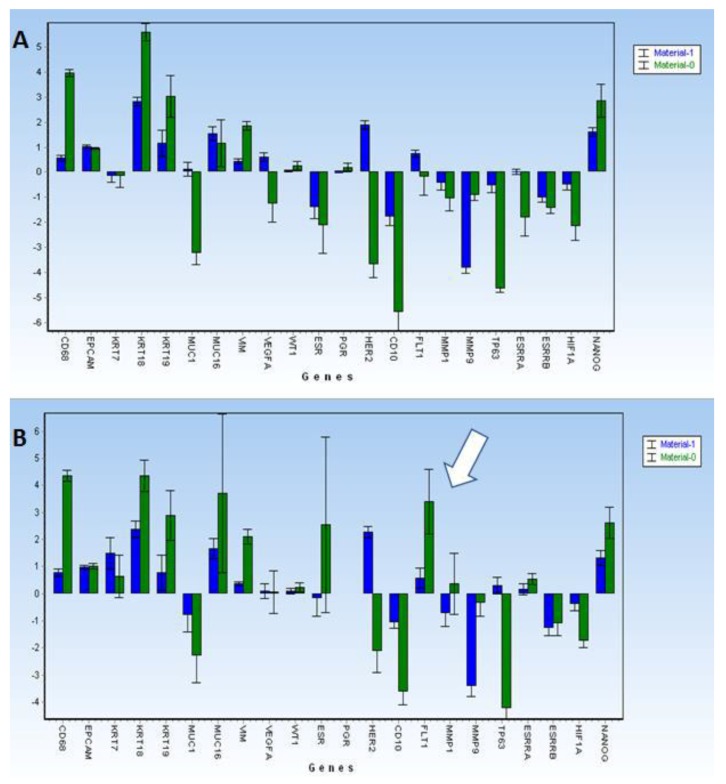
Gene expression profiling data displayed for CECs when compared to white blood cells (WBC). (**A**) Gene expression profiling data displayed for all CECs tested in the study (*n* = 52) (**B**) Gene expression profiling data for high-positive CEC (*n* = 13) samples when compared to the white blood cell fraction (WBC). In super-positive CEC samples, *FLT1* was significantly elevated (see white arrow).

The analysis of CEC samples (*n* = 52) showed that messenger RNA (mRNA) expression of the following genes was significantly elevated in the CECs fractions when compared to the WBC fractions: *KRT18*, *VIM*, *NANOG*, and *MMP9*. *KRT19* was at the limit of significance (see Figure 3A). The following genes were significantly decreased in CEC samples: *HIF1A*, *CD10*, and *MUC1*. There was a significant difference between these CEC samples and the CEC samples with high cellularity found for *FLT1* expression (see Figure 3B—white arrow).

### 3.6. CEC Prevalence and Characteristics during Menstrual Cycle (MC)

Based on the simple presumption that CECs should be present in PB during all phases of MC if they are to be used as a biomarker of the disease, blood samples from 11 patients were collected throughout all MC phases. The highest CEC numbers were detected in the after-ovulatory periods (mid-secretory phase) of MC. CECs were present throughout the MC phases but their characteristics varied. The characteristics of CECs during MC reflected the physiological cycle of endometrium decidualization. The cytomorphology of CECs captured during MC changed between epithelial, stromal, and stem cell-like.

**Figure 4:**
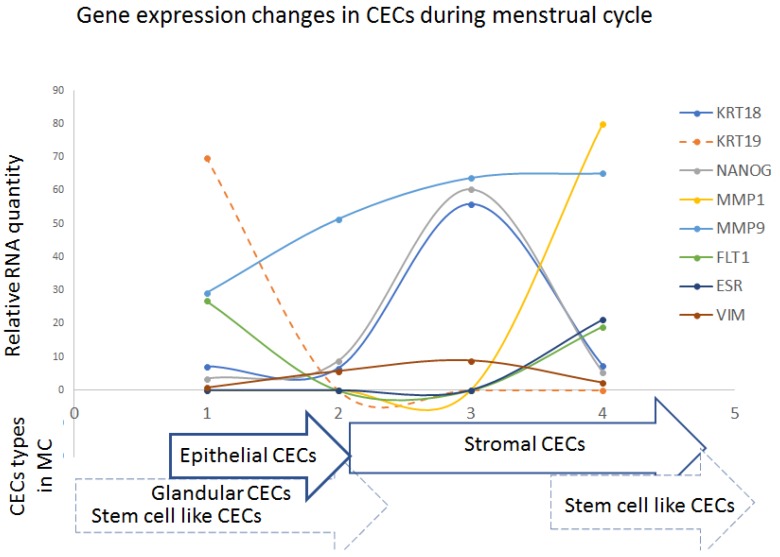
Gene expression changes in CECs during the menstrual cycle (MC; four examinations) are shown in relation to CECs cytomorphology type: epithelial, stromal, stem-cell like, glandular. Examination No. 1 (day 24 of MC), No. 2 (day seven of MC), No. 3 (day 14 of MC), No. 4 (day 21 of MC). The values are presented as relative RNA amount.

In line with this finding, gene expression changes during MC phases in CECs were analyzed, and it was observed that the structural genes like *KRT18*, *VIM*, and *NANOG* were expressed in a relatively stable manner in all four MC phases, but their expression was significantly elevated in the middle of the MC (early/mid secretory phase) (see Figure 4). In this period, the expression of *KRT18* and *VIM* increased. This could possibly represent the more frequent presence of a stem and/or mesenchymal cell population in this period. Additionally, in the late secretory and early proliferative phase, the elevation of *FLT1* and *MMP1* expression was observed.

Cells shed into circulation during the decidualization process were mostly stromal-like endometrial cells, as shown by their cytomorphology and gene expression profile. These cells were most probably estrogen or progesterone non-responsive but they did express *ESRRB*. The highest expression of *ESSRB* was found after ovulation in the secretory phase between days 20 and 26.

The *ESR1*-positive cells were regularly shed to the blood during the proliferative phases of MC (days 1–14). Epithelial *KRT19*^+^ cells which were *ESR1*-positive were typically found during this phase of MC.

## 4. Discussion

Our study confirmed the presence of CECs in most patients with histologically proven endometriosis. The CECs occurrence was confirmed during all phases of the menstrual cycle, but the CECs cytomorphology differed depending on the changing hormone levels. The cytomorphological changes were accompanied by differences in the gene expression profile, as shown by the presented data.

Subsequently, the gene expression profiling of the endometriosis lesions and of the parallel CECs samples from PB identified a range of potential biomarkers which could be used to identify CECs in patients with undiagnosed endometriosis. Early detection of CECs in women with pelvic pain or other symptoms, in addition to objective gynecological examination suspecting endometriosis, could accelerate and improve diagnosis.

The shedding of CECs into PB could be ascribed to the well-known physiology of decidualization. The process of decidualization of the uterine lining denotes the transformation of endometrial stromal fibroblasts into specialized secretory decidual cells that provide a nutritive and immune-privileged matrix essential for embryo implantation and placental development. Decidualization of the human endometrium is driven by the postovulatory rise in progesterone levels and increasing local cAMP production. In response to falling progesterone levels, spontaneous decidualization causes menstrual shedding and cyclic regeneration of the endometrium. Under endometriosis conditions, the decidualizing cells tend to be progesterone non-responsive, which results in the need for a different energy [17].

Endometrial-derived stem-cell vascular metastasis, as described in the study of Li F et al. [18], might provide a valuable explanation for cases of distant, deep infiltration and recurrent endometriosis. It was shown that circulating endometriosis stem cells propagate endometriosis through vascular dissemination and may also serve as biomarkers of active lesion establishment. Furthermore, endometriosis-derived circulating cells were consistently found in the blood of animals with endometriosis, and their number increased during new lesion establishment in the mouse endometriosis model [18]. A similar study using a mouse endometriosis model showed that donor bone marrow-derived circulating endothelial progenitor cells were found to be elevated acutely after endometriosis induction [19]. The abovementioned circulating cell types with different expression profiles may be involved in endometriosis establishment and could serve as biomarkers of active disease.

Implementing the theory of somatic stem cells, endometriosis may be regarded as a stem-cell disease [20,21]; these endometrial stem cells differentiate into local tissue types, but cells may also differentiate into the epithelium, glands, and stroma to form functional ectopic endometrial tissue [22,23]. All of the mentioned cell types were detected in different frequencies in enriched CEC samples in our study.

However, the following questions still remain: What are the characteristics of CECs causing endometriosis? What markers might be used to identify CECs with some level of certainty? The CEC-positive samples displayed elevated gene expression of *KRT18*, *KRT19*, *NANOG*, and *VIM* in most of the tested samples. The CEC cells characterized in our cohort did not express PGR (progesterone receptor) and, in at least half of the cases, *ESR1* was also not present. Does this mean that mostly hormone non-responsive CECs are shed into circulation?

The histological appearance of the endometrium was referred to as predecidua in several previous publications [18,24]. In parallel with the predecidua changes, various CEC types isolated from patients with endometriosis in our cohort showed different gene expression profiles, represented by typically elevated gene expression of *KRT18*, *NANOG*, and *VIM* or of *KRT19* and *ESR1*.

Interestingly, *KRT18*, *VIM*, and *NANOG* were elevated in the secretory phases of the menstrual cycle, while *KRT19* and *ESR1* were observed in the proliferative MC phases. Angiogenesis might be driven by elevated *FLT1* and *MMP1* in the late secretory phases. Our results mirror data reviewed by Wang et al. [16], summarizing the probable pathogenesis of endometriosis. In short, the proliferative phases of MC are presented by relatively high keratin expression, ascribed to the quickly proliferating epithelial cells. Upon epithelization, the process of lining decidualization is supported by a stem cell and/or mesenchymal cell supply. It was shown that somatic stem cells may originate from different tissue stem-cell reservoirs and/or directly from bone marrow. As shown by the distinct gene expression profiles, there were also cells with mesenchymal characteristics (*VIM*-positive) on the way to the endometrium. Similarly, circulating stromal cells (*CD10*^+^ cells) but no epithelial cells in the circulating blood of endometriosis patients were detected using another size-based separation approach (ScreenCell^®^) [25], who reported the presence of only circulating stromal cells. One of their explanations for the absence of epithelial circulating cells in their study was that cells smaller than 8 µm could have been missed using this filtration technique.

Our data showed that, during the MC, there is often elevated *KRT19* and *ESR1* detected in CECs in the proliferative phase, and that, during the whole MC, *KRT18*, *NANOG* (a stem-cell marker), and *VIM* (mesenchymal marker) are present in different levels in enriched CECs.

The results discussed in this paper offer a chance to identify CEC subtypes circulating in PB and may facilitate the management of preoperative and postoperative endometriosis therapy in the future, using the CEC characteristics and their hormone non-responsiveness. Further studies are necessary to fully understand the advantages of CEC application and its use in clinical practice.
